# Sub-Minimum Inhibitory Concentrations of Amoxicillin Modulate Biofilm Formation and the Expression of Biofilm-Associated Genes in *Enterococcus faecalis*

**DOI:** 10.3390/molecules31121986

**Published:** 2026-06-06

**Authors:** Desiye T. Tegegne, Sylwia Banaszkiewicz, Jacek Bania, Błażej Poźniak

**Affiliations:** 1 Department of Pharmacology and Toxicology, Faculty of Veterinary Medicine, Wrocław University of Environmental and Life Sciences, ul. Norwida 31, 50-375 Wroclaw, Poland; blazej.pozniak@upwr.edu.pl; 2 Animal Biotechnology Research Program, National Agricultural Biotechnology Research Center, Ethiopian Institute of Agricultural Research, Holeta P.O. Box 249, Ethiopia; 3 Department of Food Hygiene and Consumer Health Protection, Faculty of Veterinary Medicine, Wrocław University of Environmental and Life Sciences, ul. Norwida 31, 50-375 Wroclaw, Poland; sylwia.banaszkiewicz@upwr.edu.pl (S.B.); jacek.bania@upwr.edu.pl (J.B.)

**Keywords:** *Enterococcus faecalis*, biofilm formation, sub-minimum inhibitory concentration, amoxicillin, gene expression, biofilm-associated genes

## Abstract

**Background:** *Enterococcus faecalis* is one of the most frequent causes of catheter-associated urinary tract infections, largely due to its ability to form biofilms on indwelling urinary catheter surfaces, which enhance bacterial persistence and antimicrobial tolerance. Sub-minimum inhibitory concentrations (sub-MICs) of antimicrobials frequently occur in clinical settings, and growing evidence suggests that such suboptimal exposures can induce bacterial biofilm formation. We hypothesized that exposure to sub-MICs of amoxicillin, ciprofloxacin, and nitrofurantoin, antimicrobials commonly employed in the treatment of urinary tract infections, would enhance the biofilm-forming capacity of *E. faecalis* strains. **Objective:** To investigate the effects of sub-MICs of amoxicillin, ciprofloxacin, and nitrofurantoin on biofilm formation and biofilm-associated gene expression. The study focused on key biofilm-related genes, including those encoding aggregation substance protein (*asa1*), collagen adhesin (*ace*), *E. faecalis* surface protein (*esp*), gelatinase (*gelE*), cytolysin activator A (*cylA*), endocarditis antigen A (*efaA*), and the endocarditis- and biofilm-associated pili subunit A (*ebpA*) in *E. faecalis*. **Methods:** Two strains, *E. faecalis* ATCC 29212 and strain 54, were exposed to 1/8× and 1/4× MIC of amoxicillin, ciprofloxacin, and nitrofurantoin in either artificial urine medium (AUM) or tryptone soya broth (TSB). Bacterial growth kinetics were monitored by optical density measurements, while biofilm formation was quantified using a microtiter plate biofilm assay. The expression of biofilm-associated genes was analyzed using quantitative reverse transcription PCR (RT-qPCR) at 24 and 48 h following exposure to sub-MICs of amoxicillin under flow conditions mimicking the urinary tract milieu. **Results:** Exposure to sub-MICs of the three antimicrobials did not significantly affect bacterial growth in either strain or culture medium. Sub-MICs of amoxicillin significantly enhanced biofilm formation, with the most pronounced effect observed at 1/4× MIC in both AUM and TSB. In contrast, ciprofloxacin and nitrofurantoin exerted inhibitory effects on biofilm formation across both media. Gene expression analysis demonstrated time- and strain-dependent responses to amoxicillin exposure. *E. faecalis* ATCC 29212 exhibited a moderate, coordinated upregulation of adhesion- and biofilm-associated genes, particularly at 48 h. By comparison, *E. faecalis* strain 54 showed a stronger and more dynamic transcriptional response, characterized by early and sustained induction of key biofilm-related genes, including *esp* and *gelE*, as well as a pronounced late upregulation of *ebpA*. **Conclusions:** These findings emphasize the importance of maintaining therapeutically effective antimicrobial concentrations, as sub-inhibitory amoxicillin exposure may promote biofilm-associated persistence and potentially compromise treatment efficacy.

## 1. Introduction

Catheter-associated urinary tract infections (CAUTIs) represent one of the most prevalent healthcare-associated infections, accounting for up to 40% of all such infections [1,2]. The use of indwelling urinary catheters provides a surface that promotes microbial adhesion and colonization, thereby facilitating the development of persistent infections [3]. Among the uropathogens implicated in CAUTIs, *Enterococcus faecalis* has emerged as a clinically significant opportunistic pathogen, owing to its ability to adhere to urinary catheter surfaces and exhibit intrinsic and acquired resistance to multiple antimicrobial agents [4,5,6].

*Enterococcus faecalis* harbors a diverse array of virulence determinants that promote its persistence in hospital environments, facilitate transmission, and contribute to disease pathogenesis [7,8,9]. These determinants include surface adhesins that enhance attachment to host tissues and medical devices, as well as factors that support biofilm formation, enabling the organism to resist antimicrobial treatments and evade host immune responses. Additionally, the production of cytolysins and other secreted enzymes contributes to tissue damage and modulation of host immune responses, further enhancing its pathogenic potential [10,11,12]. The organism’s intrinsic and acquired resistance to multiple antimicrobials also plays a critical role in its ability to survive in clinical settings, complicating treatment strategies and promoting its emergence as a significant nosocomial pathogen [13,14,15,16]. Key biofilm genes encode microbial surface components recognizing adhesive matrix molecules (MSCRAMMs) and related factors, including aggregation substance protein (*asa1*), collagen adhesin of *E. faecalis* (*ace*), *E. faecalis* surface protein (*esp*), gelatinase (*gelE*), cytolysin activator A (*cylA*), endocarditis antigen A (*efaA*), and endocarditis- and biofilm-associated pili subunit A (*ebpA*) [7,9,17]. The expression of these biofilm-associated genes is highly dynamic and can be modulated by environmental conditions, including both the concentration and duration of antimicrobial exposure [7]. Consequently, these factors facilitate bacterial adhesion to host tissues and abiotic surfaces and play a central role in the formation of bacterial biofilms, which are structured communities of bacteria embedded within a self-produced matrix of extracellular polymeric substances (EPSs) that adhere to biotic or abiotic surfaces [9,18]. According to estimates from the NIH and CDC, up to 80% of all microbial infections and more than 60% of nosocomial infections are associated with biofilm formation [19,20,21].

Amoxicillin, ciprofloxacin, and nitrofurantoin are among the most commonly prescribed antimicrobials for the treatment of urinary tract infections [22,23]. Several studies have reported that, during antimicrobial therapy, bacteria are frequently exposed to sub-minimum inhibitory concentrations (sub-MICs) of antimicrobials due to factors such as suboptimal dosing, pharmacokinetic variability, and limited penetration into tissues or established biofilm matrices [24]. More importantly, host tissues and indwelling medical devices are exposed to a broad range of antimicrobial concentrations during the course of therapy [25]. Rather than being merely subtherapeutic and ineffective, sub-MIC levels can function as signaling cues that modulate key bacterial processes, including gene transcription, quorum sensing, and biofilm formation [26,27,28]. Such exposures act as environmental stimuli that influence bacterial physiology, shaping stress responses, biofilm development, and virulence-associated gene expression, and thereby potentially altering infection outcomes. Accordingly, a comprehensive understanding of these effects is essential for optimizing therapeutic strategies and minimizing the risk of biofilm-associated infections.

Although suboptimal antimicrobial therapy is known to elicit adaptive bacterial responses that influence physiology and pathogenicity, the effects of sub-MICs of antimicrobials on *E. faecalis* biofilm development and associated gene expression under clinically relevant flow conditions remain largely unexplored. Moreover, the majority of prior studies have utilized conventional laboratory media that do not reflect the urinary tract environment and have largely been performed under static culture conditions that do not capture the dynamic conditions of the urinary tract milieu. Importantly, our preliminary study of bacterial growth dynamics demonstrated that *E. faecalis* rapidly reached the stationary phase under static culture conditions, likely due to the swift depletion of available nutrients; this, in turn, may influence biofilm formation. Such conditions may not reflect in vivo bacterial physiology, thereby limiting the preclinical relevance of the findings for translational applications. To overcome this limitation and more accurately mimic the dynamic environment of the urinary tract, we employed a flow-based culture system using artificial urine medium. Therefore, the primary objective of this study was to evaluate the effects of sub-MICs of three commonly prescribed antimicrobials for the treatment of urinary tract infections, namely amoxicillin, ciprofloxacin, and nitrofurantoin, on bacterial growth, biofilm formation, and the expression of biofilm-associated genes in two model strains of *E. faecalis*.

## 2. Results

### 2.1. Effect of Sub-MICs of Antimicrobials on Bacterial Growth Dynamics

**Figure 1:**
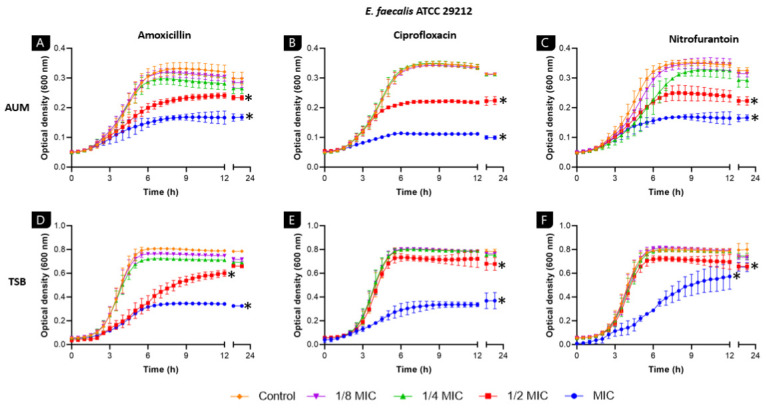
Growth curves of *E. faecalis* ATCC 29212 measured as OD_600_ over time under different antimicrobial treatments and media. Panels (**A**–**C**) show growth in AUM medium, and panels (**D**–**F**) show growth in TSB medium. The antimicrobials tested include amoxicillin (**A**,**D**), ciprofloxacin (**B**,**E**), and nitrofurantoin (**C**,**F**). Bacterial cultures were exposed to concentrations corresponding to 1×, 1/2×, 1/4×, and 1/8× the MIC, alongside untreated controls. Statistical significance was determined using a paired-sample *t*-test, including biological (*n* = 3) and technical (*n* = 3) replicates for each experiment. Asterisks denote statistically significant differences compared with the untreated control condition (*p* < 0.05).

**Figure 2:**
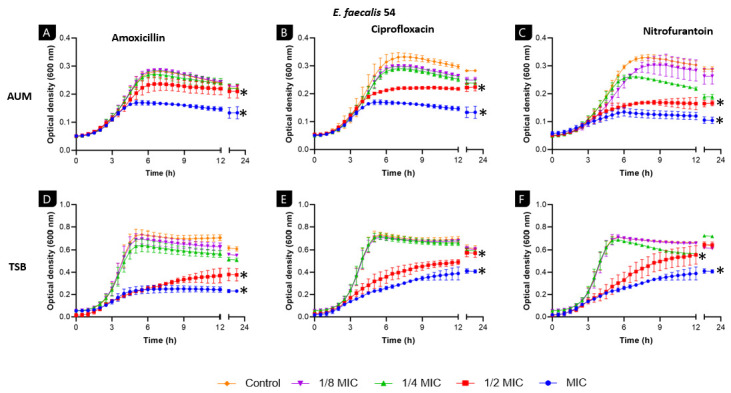
Growth curves of *E. faecalis* 54 measured as OD_600_ over time under different antimicrobial treatments and media. Panels (**A**–**C**) show growth in AUM medium, and panels (**D**–**F**) show growth in TSB medium. The antimicrobials tested include amoxicillin (**A**,**D**), ciprofloxacin (**B**,**E**), and nitrofurantoin (**C**,**F**). Bacterial cultures were exposed to concentrations corresponding to 1×, 1/2×, 1/4×, and 1/8× the MIC, alongside untreated controls. Statistical significance was determined using a paired-sample *t*-test, including biological (*n* = 3) and technical (*n* = 3) replicates for each experiment. Asterisks denote statistically significant differences compared with the untreated control condition (*p* < 0.05).

The effects of sub-MICs of amoxicillin, ciprofloxacin, and nitrofurantoin on bacterial growth were evaluated for *E. faecalis* ATCC 29212 (Figure 1A–F) and strain 54 (Figure 2A–F) in AUM and TSB. In both media, bacterial growth following exposure to sub-MIC concentrations (1/8× and 1/4× MIC) was comparable to that of the untreated control cultures, with no significant differences detected (*p* > 0.05). Conversely, treatment at MIC and 1/2× MIC resulted in significant inhibition of bacterial growth relative to the untreated controls (*p* < 0.05). These findings indicate that the tested sub-MICs (1/8× and 1/4× MIC) did not exert a significant inhibitory effect on the growth of *E. faecalis* strains during biofilm formation. Therefore, sub-MIC concentrations of 1/8× and 1/4× MIC were selected for subsequent experiments.

### 2.2. Effects of Sub-MICs of Antimicrobials on Biofilm Formation in E. faecalis

**Figure 3:**
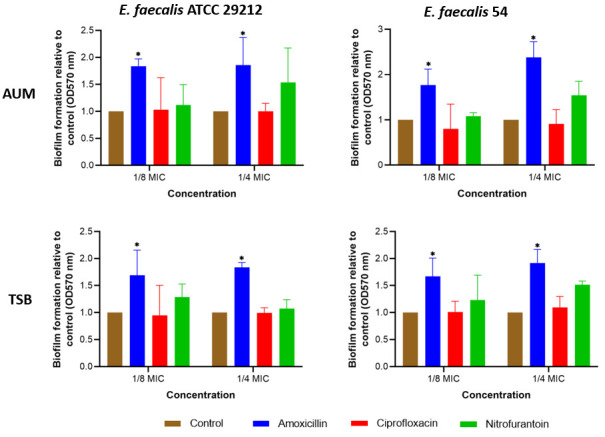
Effect of sub-MICs of amoxicillin, ciprofloxacin, and nitrofurantoin on biofilm formation by *E. faecalis*. Biofilm biomass of *E. faecalis* ATCC 29212 (**left** panels) and *E. faecalis* 54 (**right** panels) was assessed after cultivation in AUM (**top** panels) and TSB (**bottom** panels) exposed at 1/8× and 1/4× MIC of each antibiotic. Biofilm formation is expressed relative to the untreated control (set to 1.0), measured via crystal violet staining as OD570. Bars represent mean values with error bars indicating SD. Statistical significance was determined using a paired-sample *t*-test, including biological (*n* = 3) and technical (*n* = 3) replicates for each experiment. Asterisks denote statistically significant differences compared with the untreated control condition (*p* < 0.05).

Biofilm production was quantified at 1/8× and 1/4× MIC and compared with untreated controls (Figure 3). Sub-MICs of amoxicillin, ciprofloxacin, and nitrofurantoin were evaluated for their effects on biofilm formation in *E. faecalis* ATCC 29212 and *E. faecalis* 54, with increased OD_570_ values indicating enhanced biofilm formation. The hypothesis was confirmed for amoxicillin but not for ciprofloxacin or nitrofurantoin, as only sub-MIC amoxicillin exposure resulted in a significant increase in biofilm formation by *E. faecalis*. Sub-MIC exposure to amoxicillin significantly increased biofilm formation in both strains across all tested conditions. In *E. faecalis* ATCC 29212, amoxicillin treatment in both AUM and TSB resulted in elevated biofilm production at 1/8× and 1/4× MIC, with a greater effect observed at 1/4× MIC (approximately 1.8–2.0-fold relative to the control). A similar trend was observed for *E. faecalis* 54, in which biofilm formation increased under all conditions, reaching approximately 1.7-fold relative to the control at 1/8× MIC and 2.3-fold relative to the control at 1/4× MIC in AUM, with comparable increases in TSB. In contrast, sub-MICs of ciprofloxacin and nitrofurantoin exhibited inhibitory effects on biofilm formation across both the *E. faecalis* strain and growth medium. Overall, these results demonstrate that sub-MIC levels of amoxicillin enhance biofilm formation in *E. faecalis* ATCC 29212 and 54, whereas ciprofloxacin and nitrofurantoin do not induce biofilm production.

### 2.3. Expression of Genes Associated with Sub-MICs of Amoxicillin Exposure

**Figure 4:**
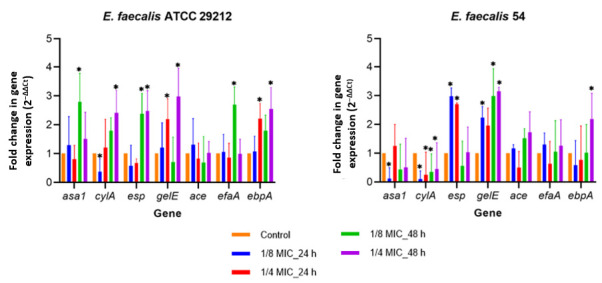
Relative expression levels of virulence and biofilm-associated genes in *E. faecalis* strains under sub-inhibitory amoxicillin pressure. Fold changes in the expression of *asa1*, *cylA*, *esp*, *gelE*, *ace*, *efaA*, and *ebpA* were evaluated in *E. faecalis* ATCC 29212 (**left**) and *E. faecalis* 54 (**right**) following exposure to 1/8 MIC and 1/4 MIC of amoxicillin for 24 h and 48 h. Gene expression levels were calculated using the 2^−ΔΔCt^ method and normalized against the untreated control group (set to 1.0) using *16S rRNA* as the internal housekeeping gene. Statistical significance was determined using one-way ANOVA, including biological (*n* = 3) and technical (*n* = 3) replicates for each experiment. Bars represent the mean fold change ± SD. Asterisks denote statistically significant differences compared with the control condition (*p* < 0.05).

Sub-MIC effects of amoxicillin on six biofilm-related genes in *E. faecalis* are shown in Figure 4. Exposure of *E. faecalis* to sub-MIC of amoxicillin led to a time- and strain-dependent modulation of biofilm- and virulence-associated gene expression. In both *E. faecalis* ATCC 29212 and strain 54, most genes showed stronger transcriptional responses after 48 h compared with 24 h, with the highest induction generally observed at 1/4× MIC. In *E. faecalis* ATCC 29212, adhesion and virulence genes, including *asa1*, *esp*, *efaA*, and *ebpA*, were significantly upregulated at 48 h, while *gelE* showed the most pronounced induction, and *cylA* increased moderately under prolonged exposure. In *E. faecalis* strain 54, the response was more variable, with early strong induction of *esp* and *gelE* at 24 h, followed by sustained or enhanced expression of *gelE* and *ebpA* at 48 h, whereas *efaA* and *asa1* showed only moderate late upregulation, and *cylA* remained weakly expressed. Overall, amoxicillin sub-MIC exposure promoted differential activation of biofilm-associated pathways depending on strain background and exposure duration.

## 3. Discussion

*Enterococcus faecalis* represents a major cause of healthcare-associated infections due to its ability to establish robust biofilms on medical devices and host tissues, creating a protective matrix that shields the bacteria from host defenses and antimicrobials, thus driving clinical persistence [29,30,31]. This complex, multifactorial process is mediated by virulence determinants, including surface adhesins, aggregation substances, and extracellular matrix components, that govern initial attachment, intercellular interaction, and biofilm maturation [32,33,34]. Given that biofilms are implicated in 60% to 80% of all microbial infections, elucidating their underlying regulatory mechanisms is critical to mitigating antimicrobial resistance and preventing therapeutic failure [35,36,37,38,39]. Importantly, sub-MICs of antimicrobials, often caused by underdosing, prolonged dosing intervals, or poor tissue penetration, can act as stressors that alter bacterial physiology and paradoxically induce biofilm formation depending on the drug, dose, and strain [40,41,42,43]. However, the impact of sub-MICs on *E. faecalis* biofilms under dynamic flow conditions in AUM remains poorly characterized. To bridge this gap in understanding biofilm-associated pathogenicity, this study evaluated the effects of sub-MIC amoxicillin, ciprofloxacin, and nitrofurantoin on *E. faecalis* biofilm formation using microtiter plate assays and analyzed corresponding alterations in gene expression via quantitative RT-PCR.

We demonstrate that exposure of *E. faecalis* ATCC 29212 and strain 54 to sub-MICs (1/8× MIC and 1/4× MIC) of tested antibiotics does not significantly affect bacterial growth in either AUM or TSB. Growth kinetics remained comparable to those of untreated controls across all time points, with no statistically significant differences observed. These findings confirm that the tested sub-MIC levels do not exert a measurable inhibitory effect on planktonic proliferation, consistent with the definition of sub-MICs as those insufficient to suppress bacterial growth [44]. Importantly, the absence of growth inhibition under these conditions supports the validity of subsequent analyses of biofilm formation, as any observed effects can be attributed to regulatory or phenotypic modulation rather than reduced cell viability. Treatment with 1/2× MIC and 1× MIC of amoxicillin and ciprofloxacin consistently and significantly inhibited bacterial growth relative to untreated controls. In contrast, both *E. faecalis* strains exposed to 1× MIC nitrofurantoin occasionally achieved final growth curves comparable to those of the control, particularly in TSB. This kinetic behavior can be explained by the definition of the MIC, which represents the threshold for inhibition of visible growth at a defined endpoint rather than complete bacterial eradication. Accordingly, following an initial drug-induced lag phase, a surviving subpopulation may have initiated cellular repair processes and subsequently resumed proliferation. This metabolic recovery is likely further facilitated by the nutrient-rich composition of TSB, which supports rapid bacterial reactivation during prolonged incubation. Additionally, nitrofurantoin is known to be light-sensitive, and exposure to light during incubation may further reduce its effective antimicrobial activity, potentially contributing to the observed regrowth dynamics.

This study shows that sub-MICs of antimicrobials, including amoxicillin, ciprofloxacin, and nitrofurantoin, differentially influence biofilm formation in *E. faecalis* ATCC 29212 and strain 54 across two growth media. The most notable result was the ability of different subinhibitory concentrations of amoxicillin to induce biofilm production in both tested *E. faecalis* strains in AUM and TSB, with 1/4× MIC consistently yielding greater biomass than 1/8× MIC and untreated controls. The pronounced biofilm induction observed following amoxicillin exposure is consistent with previous studies showing that β-lactam antibiotics, at sub-inhibitory levels, can trigger stress responses in Gram-positive bacteria such as *E. faecalis* that promote biofilm formation [39]. These responses include alterations in cell wall synthesis, increased autolysis, and the release of extracellular DNA, all of which contribute to biofilm structure and stability [26,45]. Furthermore, exposure to sub-MIC levels of β-lactams, such as amoxicillin, has been shown to upregulate genes involved in adhesion and biofilm maturation, ultimately enhancing bacterial persistence [27,28].

In contrast, ciprofloxacin and nitrofurantoin consistently exhibited inhibitory effects on biofilm formation under all tested conditions. These effects may be attributed to their distinct mechanisms of action. Ciprofloxacin targets DNA gyrase and topoisomerase IV, thereby interfering with DNA replication and related cellular processes that are essential for biofilm development, even at sub-inhibitory concentrations [46]. This moderate inhibitory effect is consistent with previous findings indicating that fluoroquinolones can reduce *E. faecalis* biofilm formation by disrupting DNA replication required for stable biofilm establishment. Importantly, nitrofurantoin, which exerts its antibacterial activity through the generation of reactive intermediates that damage multiple cellular targets, is similarly less likely to induce regulatory pathways associated with biofilm formation (37). Comparable observations have been reported in other studies, where fluoroquinolones and nitrofuran derivatives demonstrated limited biofilm-inducing potential relative to β-lactam antibiotics [26,28]. Collectively, these findings indicate the critical influence of antimicrobial class on biofilm modulation and suggest that sublethal exposure to β-lactam antibiotics, such as amoxicillin, may inadvertently enhance *E. faecalis* persistence in clinical settings. This has important therapeutic implications, particularly in environments where bacteria are frequently exposed to fluctuating or sub-inhibitory antimicrobial concentrations.

The present study reveals distinct temporal patterns of virulence-associated gene expression in *E. faecalis* ATCC 29212 and strain 54, indicating divergent adaptive and pathogenic strategies. Overall, gene expression changes were minimal at early time points but became increasingly pronounced over time, suggesting a progressive response associated with biofilm development and maturation. This pattern is consistent with previous reports showing that sub-MIC amoxicillin exposure induces cell wall stress responses in *E. faecalis*, thereby enhancing the expression of genes involved in adhesion, aggregation, and extracellular matrix production.

In *E. faecalis* ATCC 29212, the moderate and coordinated upregulation of virulence genes reflects the stable expression profile typical of *E. faecalis* strains. Adhesion-related genes such as *asa1* and *esp* were expressed at moderate levels, supporting initial attachment without inducing a strong virulence response [47,48]. The gradual increase in *gelE* and *efaA* expression at later stages is consistent with their roles in biofilm maturation and host interaction, while upregulation of *ebpA* at both times at higher concentrations supports its established role in pili formation and biofilm development during prolonged growth phases [49,50,51]. Upregulation of *cylA* indicates increased expression of the cytolysin operon, suggesting enhanced cytolysin production and a corresponding rise in bacterial virulence and cytotoxic potential [52]. In contrast, the relatively stable expression of *ace* in *E. faecalis* ATCC 29212 is also in agreement with studies suggesting that collagen-binding proteins may be constitutively expressed to maintain readiness for host interaction rather than being strongly inducible [53].

In contrast, *E. faecalis* strain 54 exhibited a markedly stronger and more dynamic transcriptional response, particularly in *esp* and *gelE*, which were highly upregulated at intermediate time points. Downregulation of *cylA* may indicate suppression of cytolysin-mediated virulence and a regulatory shift favoring biofilm-associated survival under stress conditions, consistent with the observed inverse relationship between cytolysin expression and robust biofilm formation [54,55]. This pattern is consistent with previous studies on clinical isolates, where enhanced expression of these genes has been linked to increased virulence, biofilm formation, and cytolysin production [31,56]. The elevated *esp* expression, in particular, has been widely associated with hospital-acquired strains and their ability to form persistent biofilms on medical devices. Interestingly, in strain 54, this adaptational *esp* upregulation appeared faster than in the ATCC 29212 strain. The early downregulation of *asa1* at lower amoxicillin concentrations in *E. faecalis* strain 54 suggests a regulated adhesion mechanism that may be influenced by environmental cues. Aggregation substance, encoded by the *asa1* gene, is a surface-associated protein that plays a critical role in biofilm formation and adherence to host tissues, both of which are key determinants of pathogenicity [57]. Similar expression patterns have been reported in clinical isolates, where aggregation substance is induced under specific conditions to promote cell-to-cell contact and plasmid transfer [58]. Furthermore, the significant increases in *ebpA* at later stages reinforce the transition of *E. faecalis* strain 54 into a highly adherent and biofilm-competent phenotype, supporting observations from previous research that pili-associated genes are critical for long-term colonization and infection persistence [41]. Overall, the differences observed between the two strains support the notion that *E. faecalis* strain 54 exhibits more robust and dynamic virulence gene expression compared to *E. faecalis* ATCC 29212. The latter strain appears to rely on a steady and regulated expression strategy, which may favor persistence under controlled conditions. In contrast, *E. faecalis* strain 54 demonstrates rapid activation of multiple virulence pathways, consistent with previous findings that associate clinical isolates with increased pathogenicity and adaptability [31]. It should be noted that the *gelE* upregulation in both strains may be associated with the presence of gelatin in the culture medium. Although gelatin is commonly added to media applied in biofilm studies and in cases where better reproduction of urinary viscosity is needed (flow models), it may possibly channel the bacterial adaptational response in a direction that does not fully reflect the situation seen in the urinary tract.

Although the current model seems to be much more clinically relevant as compared to the standard microtiter plate model, it is still only an in vitro model, which may not fully replicate the complex in vivo environment encountered during infection, including host immune interactions, tissue-specific factors, and pharmacokinetic antimicrobial fluctuations. Furthermore, the use of glass as a substrate may not fully reflect the adaptive response that would develop if bacteria had direct contact with tissues or specific polymer surfaces of indwelling devices. Additionally, only two *E. faecalis* strains were examined, which limits the generalizability of the findings across the broader genetic and phenotypic diversity of clinical isolates. The study also focused on a selected set of biofilm-associated genes and therefore may not capture the full scope of global transcriptional changes induced by sub-MIC antimicrobial exposure. Finally, protein-level validation of gene expression changes and direct mechanistic assessments of regulatory pathways were not performed, which could further strengthen the interpretation of the observed phenotypic effects.

## 4. Materials and Methods

### 4.1. Bacterial Strains, Culture Media, and Antimicrobials

Two *E. faecalis* model strains were used in this study: *E. faecalis* ATCC 29212, originally isolated from urinary tract infection and widely used in urinary tract infection research, was obtained from Microbiologics (St. Cloud, MN, USA), and *E. faecalis* strain 54, obtained from the Department of Food Hygiene and Consumer Health Protection, Wrocław University of Environmental and Life Sciences, Poland (clinical strain originally provided by Prof. A. Toledo-Arana, Instituto de Agrobiotecnología y Recursos Naturales, Universidad Pública de Navarra, Pamplona, Spain). The biofilm-forming potential of the *E. faecalis* strain 54 has been well characterized in previous studies [47,59]. Both strains are sensitive to amoxicillin, ciprofloxacin, and nitrofurantoin, which are the most common antibiotics used to treat urinary tract infections.

Tryptone soya broth (TSB) and cation-adjusted Mueller–Hinton broth (CAMHB) purchased from Oxoid (ThermoFisher Scientific, Basingstoke, UK) were used for routine bacterial cultivation and antimicrobial susceptibility testing, respectively. Artificial urine medium (AUM) [60], with its composition detailed in Supplementary Tables S1–S3, was employed as a physiologically relevant medium to simulate the urinary tract environment. To facilitate clinically relevant biofilm development, AUM was supplemented with 0.1% (*w*/*v*) glucose [61], a concentration that may be encountered in diabetic patients, a population particularly prone to urinary tract infections [62,63]. To eliminate the effect of glucose addition in the comparison between AUM and TSB, the latter medium was also supplemented with 0.1% (*w*/*v*) glucose. The most commonly prescribed antimicrobials for urinary tract infections, amoxicillin, ciprofloxacin, and nitrofurantoin, were included in this study and obtained from Sigma-Aldrich (Merck, Darmstadt, Germany). Antimicrobial stock solutions were prepared, diluted in accordance with Clinical and Laboratory Standards Institute (CLSI) guidelines [64], aliquoted, and stored at −20 °C. Preliminary susceptibility testing confirmed that both *E. faecalis* strains were susceptible to all three antimicrobials tested (Supplementary Table S4). Sub-MIC values used to assess the effects of antimicrobials on bacterial growth dynamics and biofilm formation were selected based on the MICs of the respective antimicrobials in their corresponding media.

### 4.2. Effect of Sub-MICs of Antimicrobials on Bacterial Growth Kinetics

This assay was performed to evaluate the potential growth-inhibitory effects of sub-MICs of amoxicillin, ciprofloxacin, and nitrofurantoin and to ensure that these sub-MIC levels did not influence biofilm formation by reducing bacterial growth in both AUM and TSB media. Briefly, overnight cultures of the two *E. faecalis* strains were diluted in fresh medium to an optical density at 600 nm (OD_600_) of 0.01 (approximately 1 × 10^6^ CFU/mL). Subsequently, 100 μL aliquots were dispensed into 96-well microtiter plates (Sarstedt, Nümbrecht, Germany). Each well was supplemented with 100 μL of antimicrobial solution at twice the desired final concentration, resulting in final concentrations of 0 (control; fresh medium only), 1/8× MIC, 1/4× MIC, 1/2× MIC, or 1× MIC in a total volume of 200 μL per well. Bacterial growth kinetics were assessed by measuring OD_600_ at 30 min intervals using a Tecan Spark microplate reader (Tecan, Männedorf, Switzerland). Positive controls consisted of untreated bacterial cultures, while negative controls contained only growth medium to account for background absorbance [65].

### 4.3. Effect of Sub-MICs of Antimicrobials on Biofilm Formation Under Static Conditions

The effect of sub-MICs of amoxicillin, ciprofloxacin, and nitrofurantoin on *E. faecalis* biofilm formation was evaluated at 1/8× and 1/4× MIC and compared with an untreated control in both media, using a previously described method with minor modifications [66]. Briefly, overnight cultures were diluted to an OD_600_ of 0.01 in fresh AUM or TSB. Subsequently, 100 μL of the bacterial suspension was dispensed into 96-well microtiter plates (Nunc™, Thermo Fisher Scientific, Roskilde, Denmark), followed by the addition of 100 μL of antimicrobial solutions prepared at twice the desired final concentration, yielding final concentrations of 0 (control; fresh medium only), 1/8× MIC, or 1/4× MIC in a total volume of 200 μL per well. Positive controls consisted of untreated bacterial cultures, while negative controls contained only growth medium to account for non-specific staining [67,68]. Plates were incubated at 37 °C for 24 h under static conditions.

After incubation, planktonic cells were carefully removed, and wells were gently washed with normal saline and air-dried. The biofilms were stained with 0.1% (*v*/*v*) crystal violet (Merck, Darmstadt, Germany) and incubated at room temperature for 15 min, and excess stain was removed by three gentle washes with sterile normal saline. After the biofilms had dried, the crystal violet was solubilized by adding 200 μL of 33% (*v*/*v*) acetic acid (Merck, Darmstadt, Germany), and the extent of biofilm biomass was quantified by measuring absorbance at 570 nm using a Tecan Spark microplate reader. Following OD_570_ measurements, relative biofilm formation was calculated as the ratio of sub-MIC antimicrobial-exposed samples to the untreated growth control, enabling quantification of inhibitory (<1), no effect (=1), or enhancing (>1) effects of antimicrobials compared with untreated bacteria.

### 4.4. Effect of Sub-MICs of Amoxicillin on Biofilm-Associated Gene Expression

#### 4.4.1. Biofilm Culture in a Drip-Flow Biofilm Reactor

**Figure 5:**
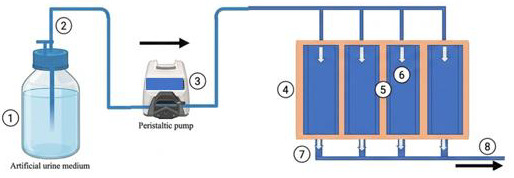
Schematic representation of the experimental biofilm flow system. The experimental perfusion system is configured as a continuous-flow setup utilizing an AUM reservoir (1), consisting of a glass bottle from which medium is drawn via an AUM inlet tube (2). A programmable peristaltic pump (3) controls the volumetric flow rate of the medium as it is driven into the drip-flow biofilm reactor (4). This reactor has four parallel channels (5) designed for biofilm growth testing on placed glass slides. Internal flow indicators (6), represented by white arrows, demonstrate the uniform downward flow of the medium within each individual chamber. After passing over the test substrates, the spent medium enters a lower collection manifold assembly (7) that pools the effluent from all four chambers before it is ultimately directed through the final effluent line/outlet tube (8) to waste. The system is maintained at a slight inclination (~10°) to ensure consistent flow and incubated at 37 °C.

Biofilms were cultivated using a drip-flow biofilm reactor (BioSurface Technologies Corp., Bozeman, MT, USA) according to the method described by Goeres et al., with minor modifications [69], as shown in Figure 5. Briefly, overnight cultures of *E. faecalis* strains were diluted to an OD_600_ of 0.01 in TSB, and 12.5 mL of the inoculum was introduced into each reactor channel containing sterile glass slides. The reactor was incubated under static conditions at 37 °C for 12 h to allow initial bacterial adhesion. Following the adhesion phase, the reactor was inclined at 10° and connected to a reservoir containing AUM, supplemented either with or without sub-MICs of amoxicillin. Amoxicillin was selected for gene expression analysis at 24 h and 48 h under sub-inhibitory conditions based on its observed ability to enhance biofilm formation at sub-MICs in static microtiter plate biofilm assays. AUM was employed in gene expression experiments to better mimic physiologically relevant conditions for studying *E. faecalis* in a uropathogenic context, whereas previous studies have largely relied on standard culture media, such as TSB. Continuous medium flow was initiated at a rate of 0.4 mL/min per channel [70] and maintained at 37 °C for up to 48 h. For time-point analyses, biofilms were harvested at 24 and 48 h. At each time point, microscope glass slides were aseptically removed from the reactor and gently rinsed with sterile normal saline to remove non-adherent cells. Biofilms were then scraped into saline and homogenized prior to downstream analyses.

#### 4.4.2. RNA Isolation

The harvested bacterial pellets were resuspended in a 100 µL buffer composed of Tris-HCl (pH 7.4; Sigma-Aldrich, Darmstadt, Germany), lysozyme (10 mg/mL; Sigma-Aldrich, Darmstadt, Germany), proteinase K (20 mg/mL; Thermo Fisher Scientific), and 10% SDS (Sigma-Aldrich, Darmstadt, Germany), followed by incubation at 37 °C for 30 min. Subsequently, 1 mL of TRI Reagent (Sigma-Aldrich, Darmstadt, Germany) was added and mixed well, and the samples were stored at −80 °C overnight. The samples were then thawed at room temperature, after which 200 µL of pure chloroform (Sigma-Aldrich, Warsaw, Poland) was added. Mechanical disruption of bacterial suspension with 425–600 µm glass beads (Sigma-Aldrich, Poland) was performed using a TissueLyser LT (Qiagen, Hilden, Germany) for three cycles of 2 min agitation, each followed by a 1 min incubation on ice. The samples were then incubated at room temperature for 5 min to allow phase separation. Following centrifugation at 12,000× *g* for 15 min at 4 °C, the upper aqueous phase (500 µL) was carefully transferred to a new microcentrifuge tube, and an equal volume of pure isopropanol (Chempur, Piekary Śląskie, Poland) was added. The mixture was incubated at −20 °C for 20 min and subsequently centrifuged at 12,000× *g* for 10 min at 4 °C. The supernatant was discarded, and the pellet was washed with 1 mL of 70% ethanol (Chempur, Poland). After incubation at −20 °C for 20 min, samples were centrifuged at 7600× *g* for 5 min at 4 °C. The washing and centrifugation steps were repeated twice. The resulting pellet was air-dried at room temperature for 7 min and resuspended in 50 µL of RNase-free water (Invitrogen, Waltham, MA, USA).

#### 4.4.3. Reverse Transcription and Quantitative Real-Time PCR

Complementary DNA (cDNA) was synthesized using the iScript™ cDNA Synthesis Kit (Bio-Rad, Hercules, CA, USA) according to the manufacturer’s instructions and subsequently stored at −20 °C until further analysis. Transcriptional levels of biofilm-associated genes were quantified by qRT-PCR following previously described protocols [71,72]. Briefly, each 20 μL reaction consisted of 12.5 μL of 2× SsoFast™ EvaGreen^®^ Supermix (Bio-Rad, USA), 1 μL of cDNA template, and 0.5 μL each of forward and reverse primers (20 µM), with nuclease-free water added to reach the final volume. Amplifications were performed using a QuantStudio™ 5 Real-Time PCR System (Applied Biosystems, Waltham, MA, USA). The target genes, corresponding primer sequences, and expected amplicon sizes are listed in Table 1. Thermal cycling conditions consisted of an initial denaturation step at 98 °C for 2 min, followed by 40 cycles of denaturation at 98 °C for 15 s and annealing/extension at 60 °C for 30 s. A melting curve analysis was performed to confirm the specificity of the amplification. All reactions were performed in technical duplicates across three independent biological replicates. To rule out the presence of genomic DNA in the RNA preparations, control reactions were performed in which reverse transcriptase was omitted. Amplification efficiency for each target gene was determined using standard curves generated from serial five-fold dilutions of genomic DNA. All primer pairs exhibited efficiencies greater than 98%, as calculated from the slope of the standard curves according to the following equation: E(%) = (10^−(1/slope)^ − 1) × 100 [73].

The mRNA expression levels were quantified relative to the endogenous reference gene *16S rRNA* [74]. Relative gene expression was calculated using the 2^−ΔΔCt^ method [75], where values >1 indicate upregulation and values <1 indicate downregulation [66,76].

### 4.5. Statistical Analysis

Statistical analyses were performed using GraphPad Prism (version 9.0.0; Boston, MA, USA). Data normality was assessed using the Shapiro-Wilk test, and all data were found to be normally distributed. Following this evaluation, all subsequent statistical analyses were conducted. All experiments were conducted in triplicate on three independent days using separate cultures, and results are presented as mean ± standard deviation (SD). Differences between treated and untreated groups for each antimicrobial concentration, with respect to biofilm formation and gene expression, were assessed using a paired-sample *t*-test. Additionally, one-way ANOVA followed by Dunnett’s multiple-comparison test was applied to compare gene expression levels across treatment concentrations with the control group. A *p*-value < 0.05 was considered statistically significant.

## 5. Conclusions

Our study demonstrates that sub-MIC exposure to amoxicillin consistently and significantly enhanced biofilm formation in *E. faecalis* ATCC 29212 and *E. faecalis* 54, accompanied by the strongest and most widespread upregulation of adhesion-, biofilm-, and virulence-associated genes, particularly at later time points. In contrast, ciprofloxacin and nitrofurantoin did not promote biofilm development and generally exhibited inhibitory effects. Notably, strain-dependent differences in gene expression responses further highlighted the greater adaptive and virulence potential of *E. faecalis* 54 compared with *E. faecalis* ATCC 29212. Overall, these findings demonstrate that sublethal exposure to amoxicillin can stimulate biofilm formation and virulence gene expression in the studied *E. faecalis* strains, highlighting the importance of maintaining effective antimicrobial concentrations during therapy and its potential role in bacterial persistence.

## Figures and Tables

**Table 1:** qRT-PCR primer sets designed for the determination of transcript levels of biofilm-associated genes.

Gene	Encoded Product	Primers Sequence (5′→3′)	Amplicon Size (bp)	References
*16S rRNA*	16S ribosomal RNA	16SF: GTGAGGTAACGGCTCACCAAG 16SR: TGTCTCAGTCCCAGTGTGGC	76	This study
*asa1*	Aggregation substance protein	asaF: AAACACCCGCTACACCAGAA asaR: GGCGTAGCATCTGTTGGGAT	70	This study
*gelE*	Gelatinase	gelF: GCTCCGATTCCAGCAGAGTT gelR: AGTGATGCTCGTGATGCGAT	101	This study
*ace*	Collagen-binding adhesin *E. faecalis*	aceF: CGGATCGACAAGGAAGTGGT aceR: CCTTGTTGCTCAAACTCGGC	109	[71]
*cylA*	Cytolysin activator A	cylF: CCACTAGGCAAAGCTGCTGA cylR: AGCTGCGCTTACTTCTGGAG	58	[71]
*ebpA*	Endocarditis and biofilm-associated pili subunit A	ebpF: CGTTTCAGCCATTAGCCACG ebpR: CTTCACGCCAGGTGCTTTTC	74	[71]
*efaA*	*E. faecalis* antigen A	efaF: CCGTTACCAGAAGACATTGCG efaR: TAAACCAGCCATTTCCGCCT	91	[71]
*esp*	Enterococcal surface protein	espF: GCATCAGTATTAGTTGGT espR: TTCCTTGTAACACATCAC	196	[72]

## Data Availability

The original contributions presented in this study are included in the article/Supplementary Material. Further inquiries can be directed to the corresponding authors.

## Supplementary Materials

The following supporting information can be downloaded at: https://www.mdpi.com/article/10.3390/molecules31121986/s1, Table S1: Composition of AUM; Table S2: Components of 5× concentrated Calcium chloride/urea stock solution; Table S3: Final working concentration of artificial urine; Table S4: Minimum inhibitory concentrations of amoxicillin, ciprofloxacin, and nitrofurantoin against two *E. faecalis* strains in two different media.
