# Supplementary material for: Sub-Minimum Inhibitory Concentrations of Amoxicillin Modulate Biofilm Formation and the Expression of Biofilm-Associated Genes in Enterococcus faecalis
Source: Molecules. 2026 Jun 6;31(12):1986. doi: 10.3390/molecules31121986 (PMC13305534; doi:10.3390/molecules31121986)
Supplement: Supplementary file 1 [file molecules-31-01986-s001.zip › molecules-4348855-supplementary.pdf]

Supplementary material

1. Supplementary Tables

Table S1. Composition of AUM [1]

Artificial urine solution 1

Component	Mass (gram)
Magnesium chloride hexahydrate ($MgCl_2.6H_2O$)	3.25
Sodium chloride (NaCl)	23
Sodium sulfate (Na_2SO_4)	11.5
Trisodium citrate ($Na_3C_6H_5O_7.2H_2O$)	3.25
Disodium oxalate ($2Na_2C_2O_4$)	0.1
Potassium dihydrogen phosphate (KH_2PO_4)	14
Potassium chloride (KCl)	8
Ammonium chloride (NH_4Cl)	5
Gelatine	25
Tryptone soya broth (TSB)	2.5
Glucose	1

Initially dissolve compounds in 850 mL of deionized water, adjust the pH to ~ 5.7 using NaOH or HCl as appropriate, adding dropwise to prevent precipitation, then bring to a final volume of 1 L by addition of deionized water. Adjusting the pH to ~5.7 at this stage is very important to ensure that the final reconstituted and dilute for use in the model. This solution should be **sterilized by autoclaving**, and then store for up to three months at room temperature**.**

Artificial urine solution 2

Calcium chloride/urea stock solution at 5X concentration prepare

Table S2. Components of 5X concentrated Calcium chloride/urea stock solution

**Component**	**Mass (gram)**
Urea	125
Calcium chloride	3.25

Dissolve urea and calcium chloride in 400 mL of sterilized deionized water. These components cannot be autoclaved and must be sterilized by filtration through 0.2 μm pore sized filters. Once sterilized, this solution can be stored for up to three months at room temperature. Final working concentration of artificial urine (5 L) prepared according to the following table

Table S3. Final working concentration of artificial urine.

**Component**	**Volume (L)**
5X concentrated AU stock (Solution 1)	1.0
5X concentrated Calcium chloride/urea stock (Solution 2)	0.4
Sterilized deionized water	3.6
**Total volume**	**5**

Table S4: Minimum inhibitory concentrations of amoxicillin, ciprofloxacin, and nitrofurantoin against two *E. faecalis* strains in two different media

Strain	Medium	Antimicrobial agent (mg/L)		
		Amoxicillin	Ciprofloxacin	Nitrofurantoin
***E. faecalis* ATCC 29212**	CAMHB	0.5	0.25	8
	AUM	1	0.5	16
***E. faecalis* 54**	CAMHB	0.5	0.5	8
	AUM	1	0.5	16

References

1. Nzakizwanayo, J.; Pelling, H.; Milo, S.; Jones, B.V.: An in vitro bladder model for studying catheter-associated urinary tract infection and associated analysis of biofilms. In *Proteus mirabilis: Methods and Protocols*; Springer: Berlin/Heidelberg, Germany, 2019; pp. 139–158. https://doi.org/10.1007/978-1-4939-9601-8_14.

.
